# Supplementary material for: A comprehensive platform for analyzing longitudinal multi-omics data
Source: Nat Commun. 2023 Mar 27;14:1684. doi: 10.1038/s41467-023-37432-w (PMC10041512; doi:10.1038/s41467-023-37432-w)
Supplement: Supplementary file 1 — Supplementary Information [file 41467_2023_37432_MOESM1_ESM.pdf]

Supplementary Figures

Supplementary Figure. 1

a

Donor	Sex	Age Group	Week									
			1	2	3	4	5	6	7	8	9	10
PTID1	female	36-40	CP	CP	CP	CP	CP	CP	CP	CP	CP	CP
PTID2	male	36-40	CP	CPFRA	CPFRA	CPFRA	CPFRA	CPFRA	CPFRA	CP	CP	CP
PTID3	female	21-25	CP	CP	CP	CP	CP	CP	CP	CP	CP	CP
PTID4	male	31-35	CP	CPFRA	CPFRA	CPFRA	CPFRA	CPFRA	CPFRA	CP	CP	CP
PTID5	female	26-30	CP	CPFRA	CPFR	CPFRA	CPFR	CPFR	CPFRA	CP	CP	CP
PTID6	male	36-40	CP	CPFRA	CPFR	CPFRA	CPFR	CPFR	CPFRA	CP	CP	CP

Assay symbols: C – complete blood count, P – proteomics, F – flow cytometry, R – scRNA-seq, A – scATAC-seq

b

Dataset	Sample type	Condition(s)	Data type	Subjects (n)	Samples per subject	Analysis
Hoffman and Schadt[19, #]	Three human tissues	unknown	Simulated expression of 200 genes	25	4	VDA
Servaas et al.[26] (GSE156980)	Sorted $CD4^+$ & $CD8^+$ non-naive T-cells	Systemic sclerosis	TCRβ sequencing	4	3	VDA
Zhu et al.[3] (CNP0001102)	Human PBMC	Normal, influenza, COVID-19	scRNA-seq	5	2-5	sUMAP, VDA, SPECT, TCA
Lee et al.[2] (GSE149689)	Human PBMC	Normal, influenza, COVID-19	scRNA-seq	17	1-2	sUMAP
Hao et al.[16] (GSE164378)	Human PBMC	HIV vaccine	scRNA-seq in CITE-seq	8	3	sUMAP
Ximerakis et al.[36] (GSE129788)	Mouse brain tissue	aging	scRNA-seq	16	1	VDA, SPECT, sUMAP

#The dataset is described in "Tutorial on using variancePartition" at https://bioconductor.org/packages/release/bioc/html/variancePartition.html (accessed on September 9, 2022).

c

Software	Comparison
variancePartition	• Similar method and almost identical results<br>• PALMO can handle missing data but variancePartition cannot
tcR	• tcR is specific to TCR sequencing data, provides sample-level analysis or treats clonotype data as binary<br>• PALMO handles broad omics data types, including TCR data, and treats clonotype data as continuous
Seurat DEG	• Seurat requires users to select two contrast groups in DEG analysis and thus is inappropriate for longitudinal data with >2 timepoints<br>• PALMO treats time as a continuous variable for data with >2 timepoints or is similar to Seurat for data with 2 timepoints

Supplementary Fig. 1: Datasets used to evaluate PALMO performance. a Characteristics (sex and age group) of six healthy donors in a longitudinal study of ten weeks and specific data modalities collected on their samples. **b** Six external datasets used to evaluate PALMO. **c** Summary of benchmarking comparison between PALMO and variancePartition, tcR, and Seurat (DEG analysis). PowerPoint (version 16.69; https://www.microsoft.com/en-us/microsoft-365/powerpoint) was used to create the figure.

Supplementary Figure. 2

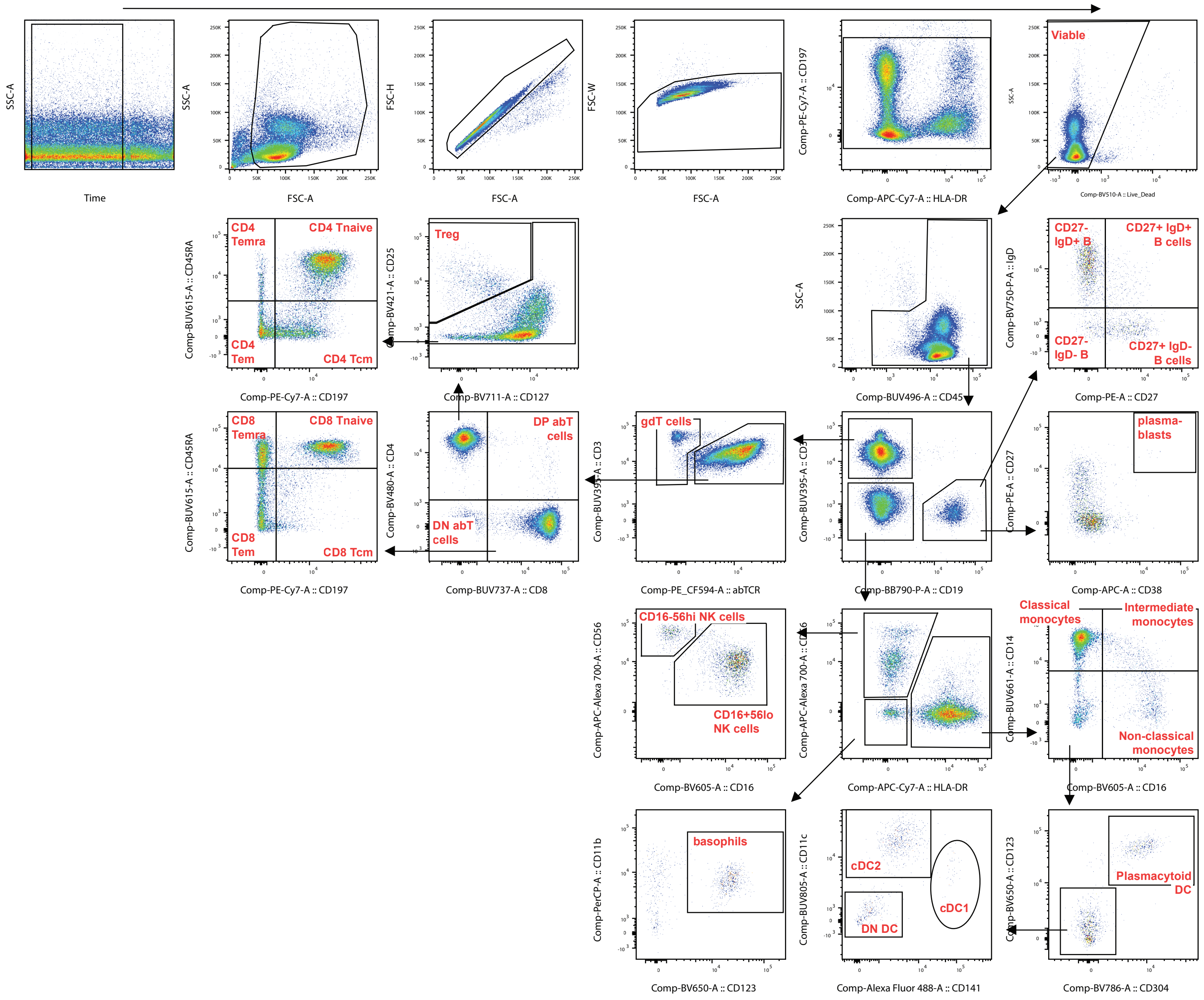

Supplementary Fig. 2: Flow cytometry gating schemes. PBMC were assayed by flow cytometry using the panel detailed in Supplementary Table 1c. Data were quantified by manual gating and summarized in Supplementary Figure 3c and Supplementary Figure 4c-d. Red labels indicate the final gates used to determine population frequencies, as applied to all samples. FlowJo (version 10.6.1; https://www.flowjo.com/) was used to generate the plots. Adobe Illustrator (version 25; https://www.adobe.com/products/illustrator.html) was used to create the figure.

Supplementary Figure. 3

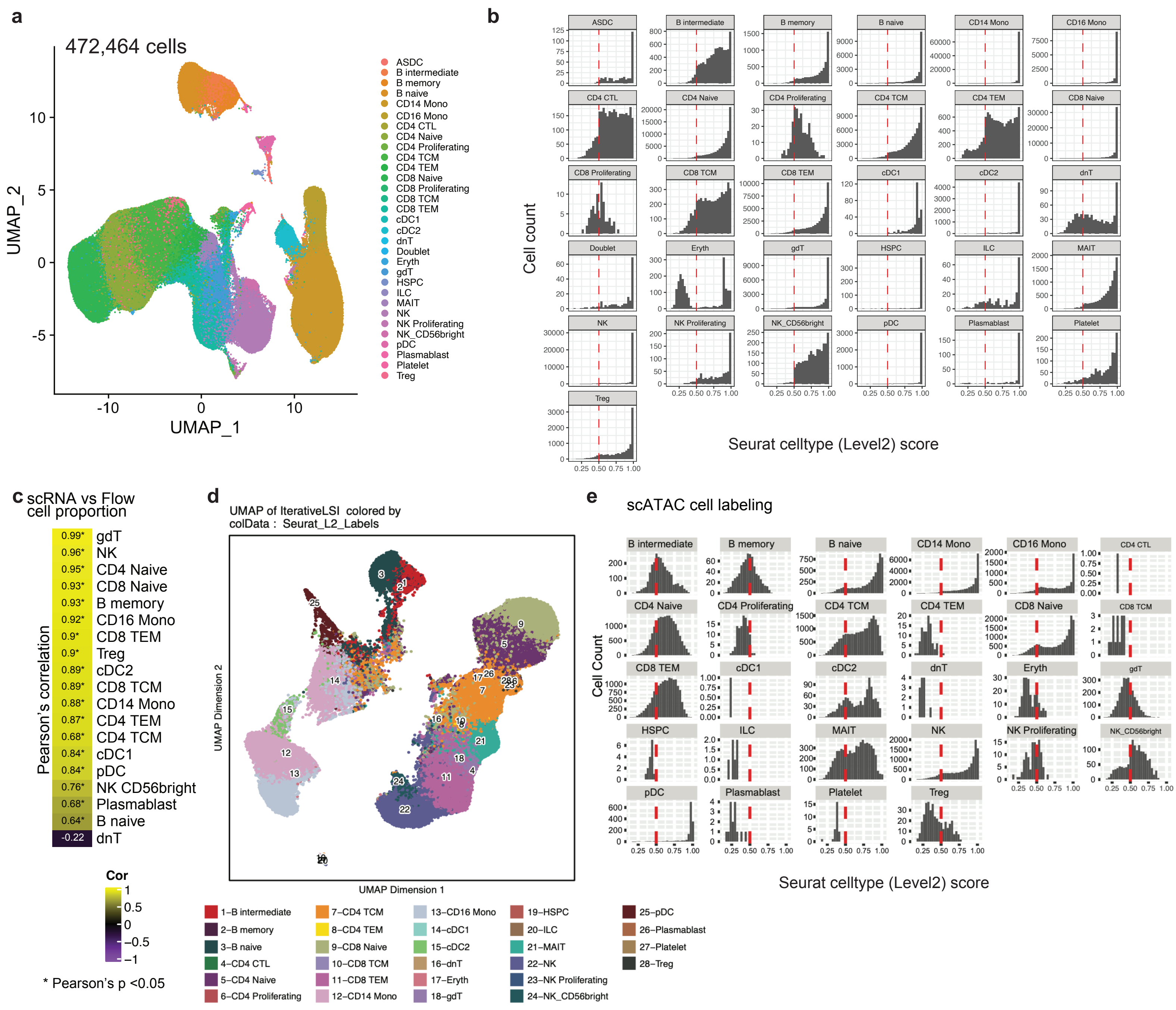

Supplementary Fig. 3: Longitudinal scRNA-seq data and scATAC-seq data on PBMCs of four healthy participants over six weeks. a Uniform Manifold Approximation and Projection (UMAP) of scRNA-seq data consisting of 472,464 peripheral blood mononuclear cells (PBMCs). The dot color represents identified cell types based on Seurat level2 labelling. **b** Distributions of labeling scores of individual cell types as observed in scRNA-seq data. Cells having scores below the red vertical dashed lines (0.5) were filtered out from analysis due to poor labeling quality. **c** Pearson correlations between frequencies of the same cell types as measured by scRNA-seq or flow cytometry on all samples. The correlation was based on 24 independent PBMC samples (6 independent samples from each of n=4 participants). The p value (two sided) was calculated using R function "cor.test()". The exact p values are given in Supplementary Data 3b. **d** UMAP projection of scATAC-seq data using iterative latent semantic indexing (LSI) for clustering and Seurat algorithm for cell labeling, as implemented in ArchR. **e** Distributions of labeling scores of individual cell types as observed in scATAC-seq data. Cells having scores below the red vertical dashed lines (0.5) were filtered out from analysis due to poor labeling quality. Adobe Illustrator (version 27.1.1; https://www.adobe.com/products/illustrator.html) was used to arrange panels and edit text.

Supplementary Figure. 4

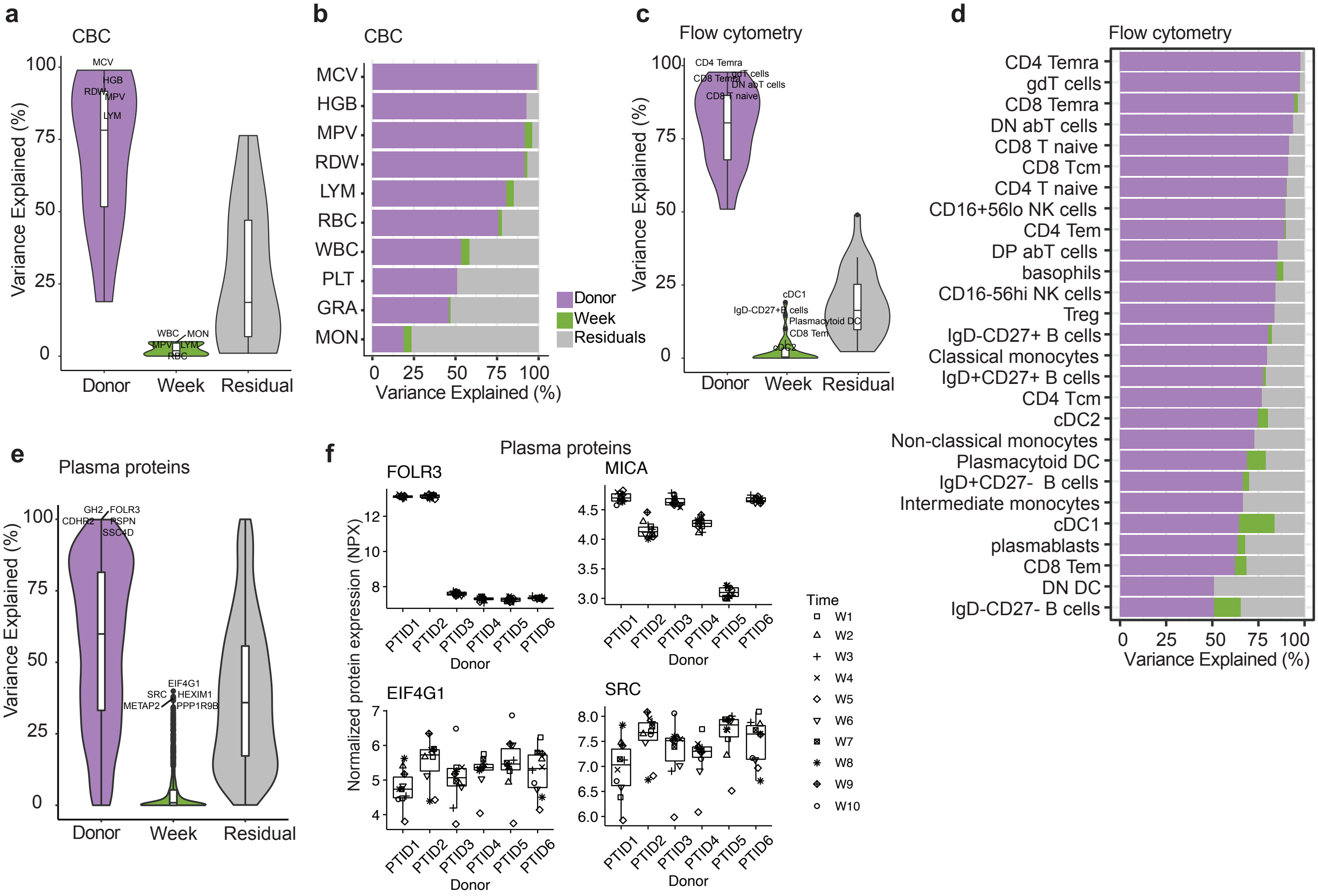

Supplementary Fig. 4: Variance decomposition on bulk longitudinal data. a Overall distributions of total variance explained by inter-donor variations (Donor), longitudinal intra-donor variations (Week) or residual variations (Residual) based on 10 complete blood count (CBC) measurements on 60 independent blood samples, one sample a week from n=6 healthy participants over 10 weeks. **b** Variance of specific CBC measurements that was explained by Donor, Week or Residual. **c** Overall distributions of total variance explained by Donor, Week or Residual based on frequencies of 27 cell types as measured by flow cytometry on 24 independent peripheral blood mononuclear cell (PBMC) samples, one sample a week from n=4 healthy participants over 6 weeks. **d** Variance of specific PBMC frequencies that was explained by Donor, Week or Residual. **e** Overall distributions of total variance explained by Donor, Week or Residual based on the abundance of 1,042 proteins as measured on 60 independent plasma samples, one sample a week from n=6 healthy participants over 10 weeks. **f** Examples of proteins whose total variance was most explained by inter-donor variations (top panel) or intra-donor variations (bottom panel). Here each boxplot contains the n=10 datapoints from the corresponding participant. Each boxplot displays the median (centerline), the first and third quartiles (the lower and upper bound of the box), and the 1.5x interquartile range (whiskers) of the data. Adobe Illustrator (version 27.1.1; https://www.adobe.com/products/illustrator.html) was used to arrange panels and edit text.

Supplementary Figure. 5

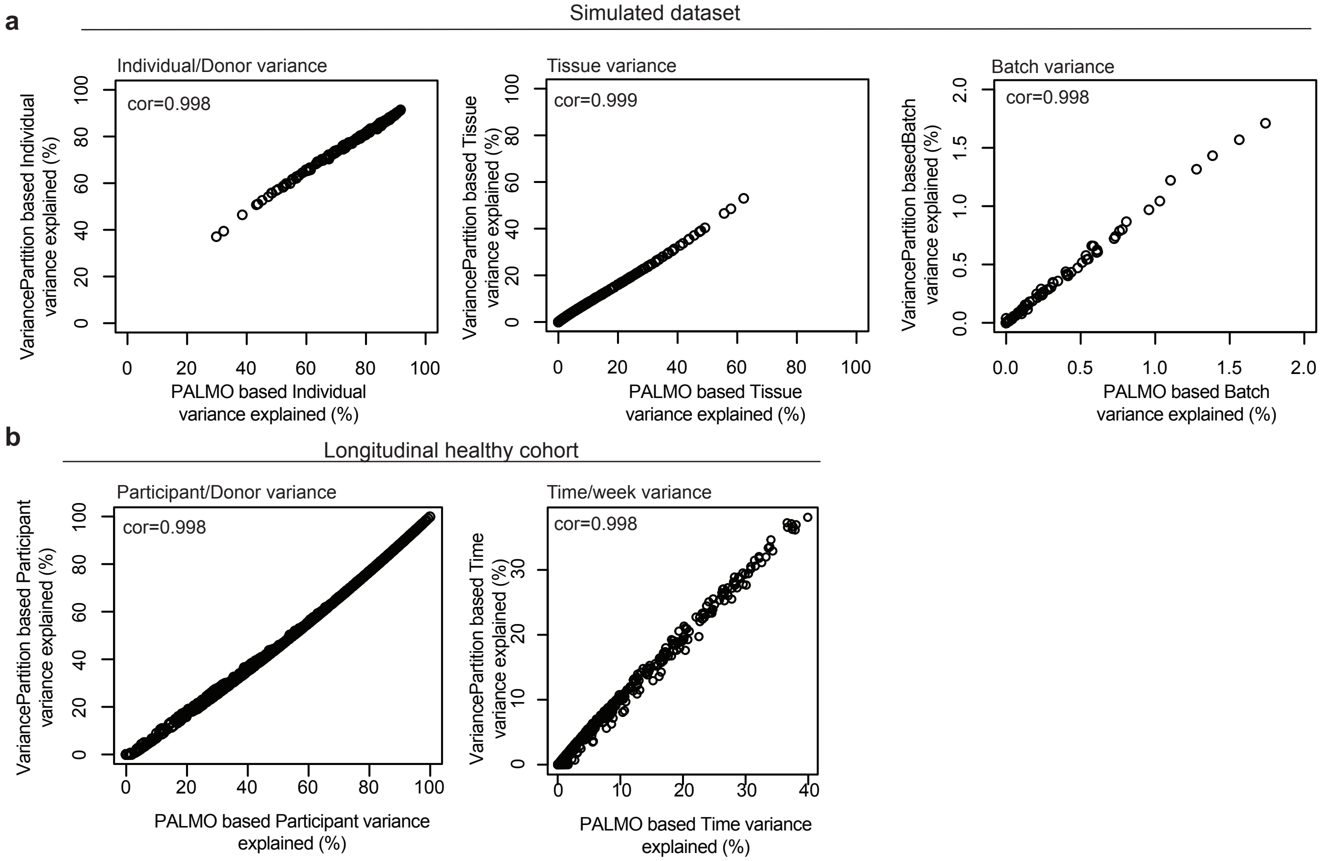

Supplementary Fig. 5: Comparison between variance decomposition analysis (VDA) and variancePartition.
a Scatter plots of percentage of total variance explained by donor (left panel), tissue (middle panel), or batch (right panel) as obtained by using VDA or variancePartition[19]. The simulated dataset of 200 genes in 100 samples of 25 donors is described in “Tutorial on using variancePartition” at https://bioconductor.org/packages/release/bioc/html/variancePartition.html (accessed on September 9, 2022). **b** Scatter plots of percentage of total variance explained by donor (left panel) or time (right panel) as obtained by using VDA or variancePartition on our longitudinal proteomics data of 60 independent samples, one sample per week from n=6 participants over 10 weeks, after removing 922 proteins with missing values out of 1,042 measured proteins. Adobe Illustrator (version 27.1.1; https://www.adobe.com/products/illustrator.html) was used to arrange panels and edit text.

Supplementary Figure. 6

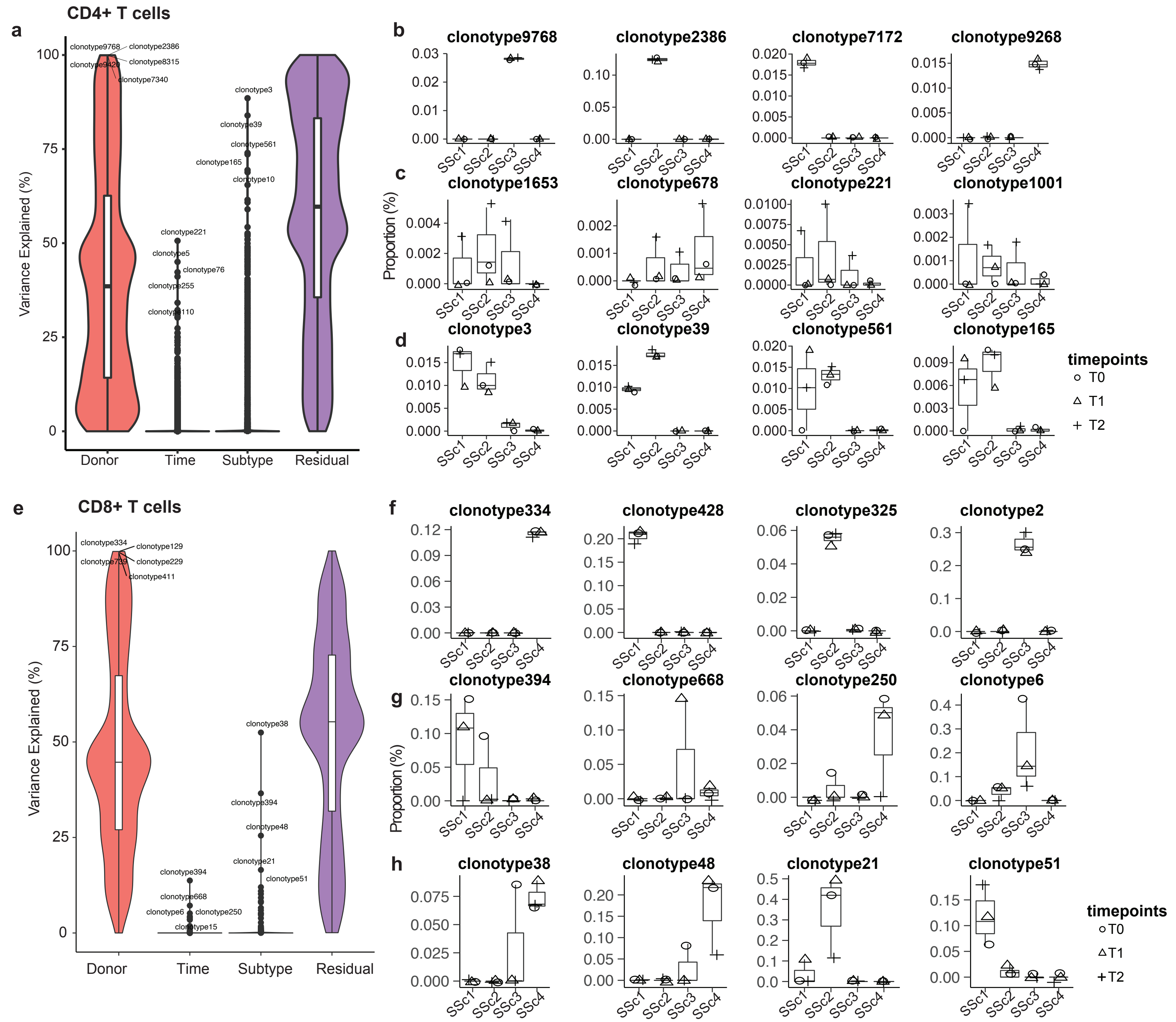

Supplementary Fig. 6: Variance decomposition on T-cell receptor (TCR) sequencing data. a Overall distributions of total variance explained by inter-donor variations (Donor), longitudinal intra-donor variations (Time), inter-subtype variations (Subtype), or residual variations (Residual) based on sequencing data of TCR β-chains from sorted CD4+ T cells of n=4 systemic sclerosis (SSc) donors, each contributing 3 independent samples over more than two years. A total of 288,597 unique clonotypes were evaluated. The two SSc subtypes considered are limited SSc and diffuse SSc. **b-d** Examples of clonotypes showing most **b** inter-donor variations, **c** intra-donor variations, or **d** inter-subtype variations. Each boxplot contains the n=3 datapoints from the corresponding donor. **e** Same as **a** but for TCRβ data of the corresponding CD8+ T cells. A total of 11,739 unique clonotypes were evaluated. **f-h** Same as **b-d** but for TCRβ data of the corresponding CD8+ T cells. Each boxplot displays the median (centerline), the first and third quartiles (the lower and upper bound of the box), and the 1.5x interquartile range (whiskers) of the data. Adobe Illustrator (version 27.1.1; https://www.adobe.com/products/illustrator.html) was used to arrange panels and edit text.

Supplementary Figure. 7

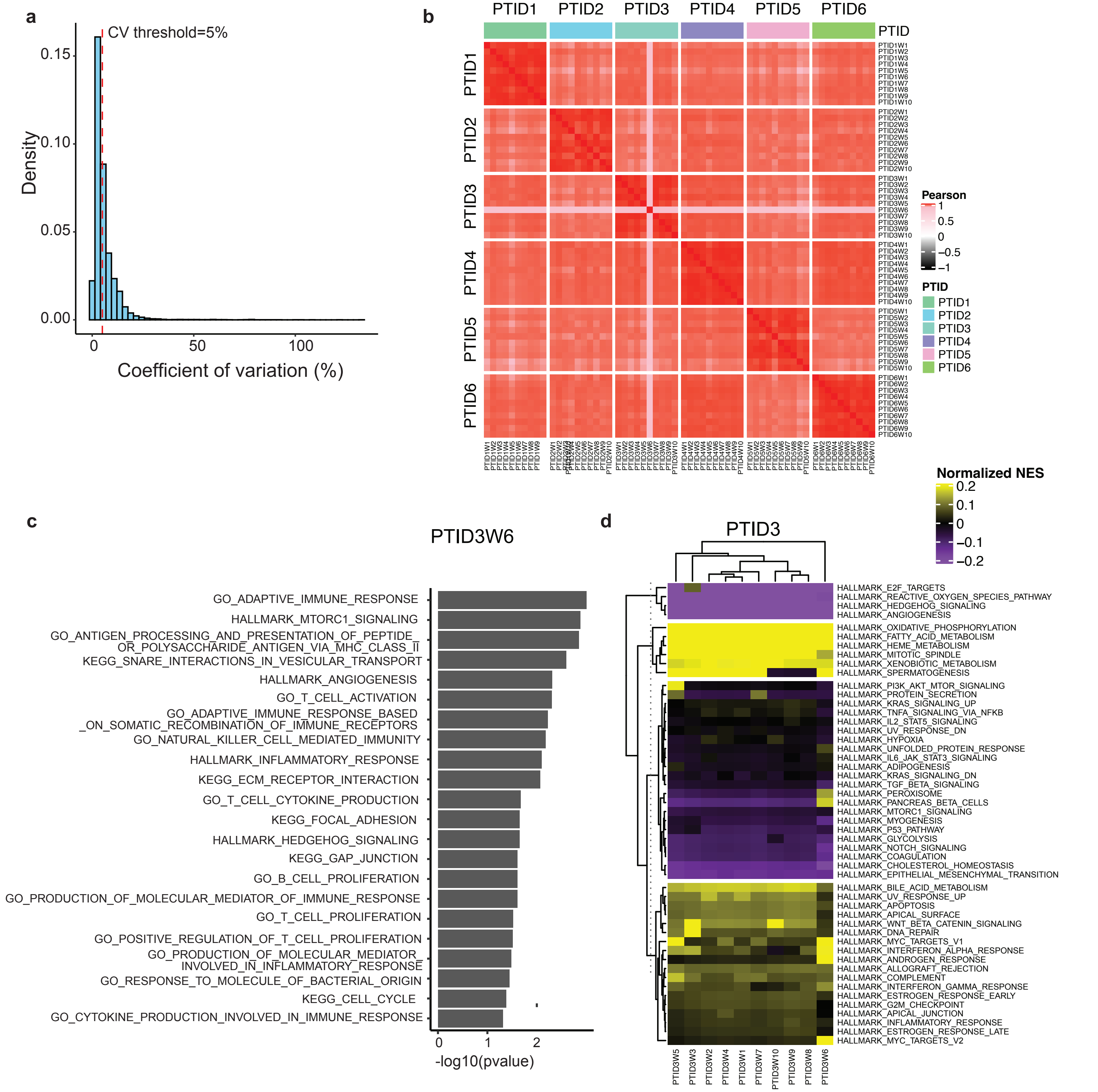

Supplementary Fig. 7: Coefficient of variation (CV) profiling (CVP) of longitudinal plasma proteomics data. **a** Histogram of coefficient of variation (CV) of normalized protein expression (NPX) as measured on 1,042 proteins over 10 independent plasma samples from each of n=6 participants. CV of 5% was selected as the cutoff separating longitudinally stable versus variable proteins. **b** Heatmap showing NPX intra- and inter-donor correlations. The correlation between any sample pair was calculated based on the 1,042 measured proteins. **c** Top pathways (p<0.05) from gene set enrichment analysis (GSEA) on outlier proteins detected in donor PTID3 at week 6. The p value was based on two-sided Fisher's exact test. **d** Single-sample GSEA (ssGSEA) on outlier proteins, showing enrichment in MYC targets, IFN-alpha response, etc., at week 6. Adobe Illustrator (version 27.1.1; https://www.adobe.com/products/illustrator.html) was used to arrange panels and edit text.

Supplementary Figure. 8

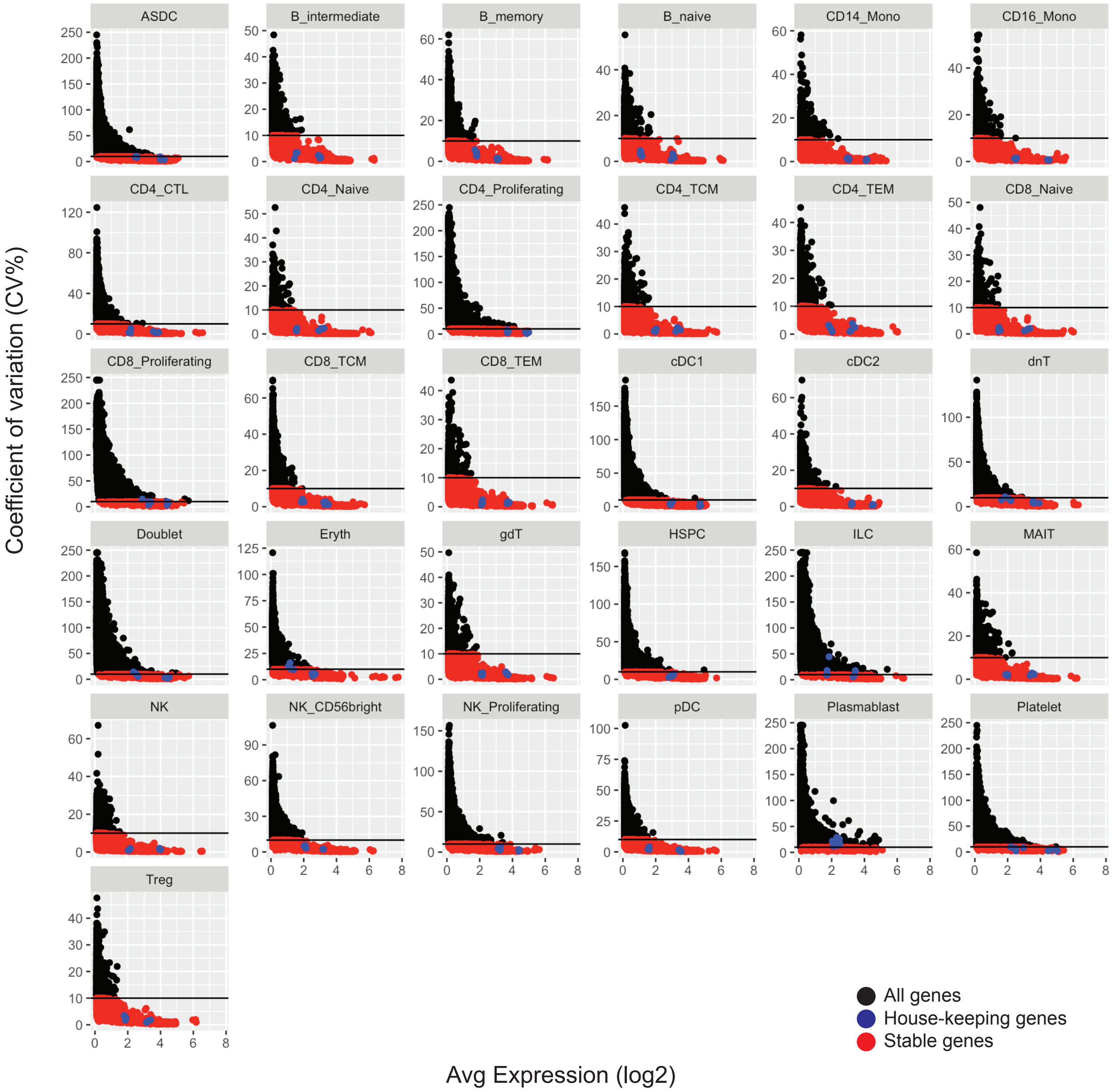

Supplementary Fig. 8: Scatter plots of coefficient of variation (CV) of longitudinal scRNA-seq data of individual cell types. Scatter plots of CV versus mean of gene expression (log2(avg counts)) as measured on 6 independent peripheral blood mononuclear cell (PBMC) samples from each of n=4 participants. Only reliable genes with an average expression ≥0.1 were kept. Results from individual participants were calculated separately and combined. Housekeeping genes ACTB and GAPDH (blue) were used to select a CV threshold of 10% by which genes were split into longitudinally stable (red) or variable (black). Adobe Illustrator (version 27.1.1; https://www.adobe.com/products/illustrator.html) was used to arrange panels and edit text.

Supplementary Figure. 9

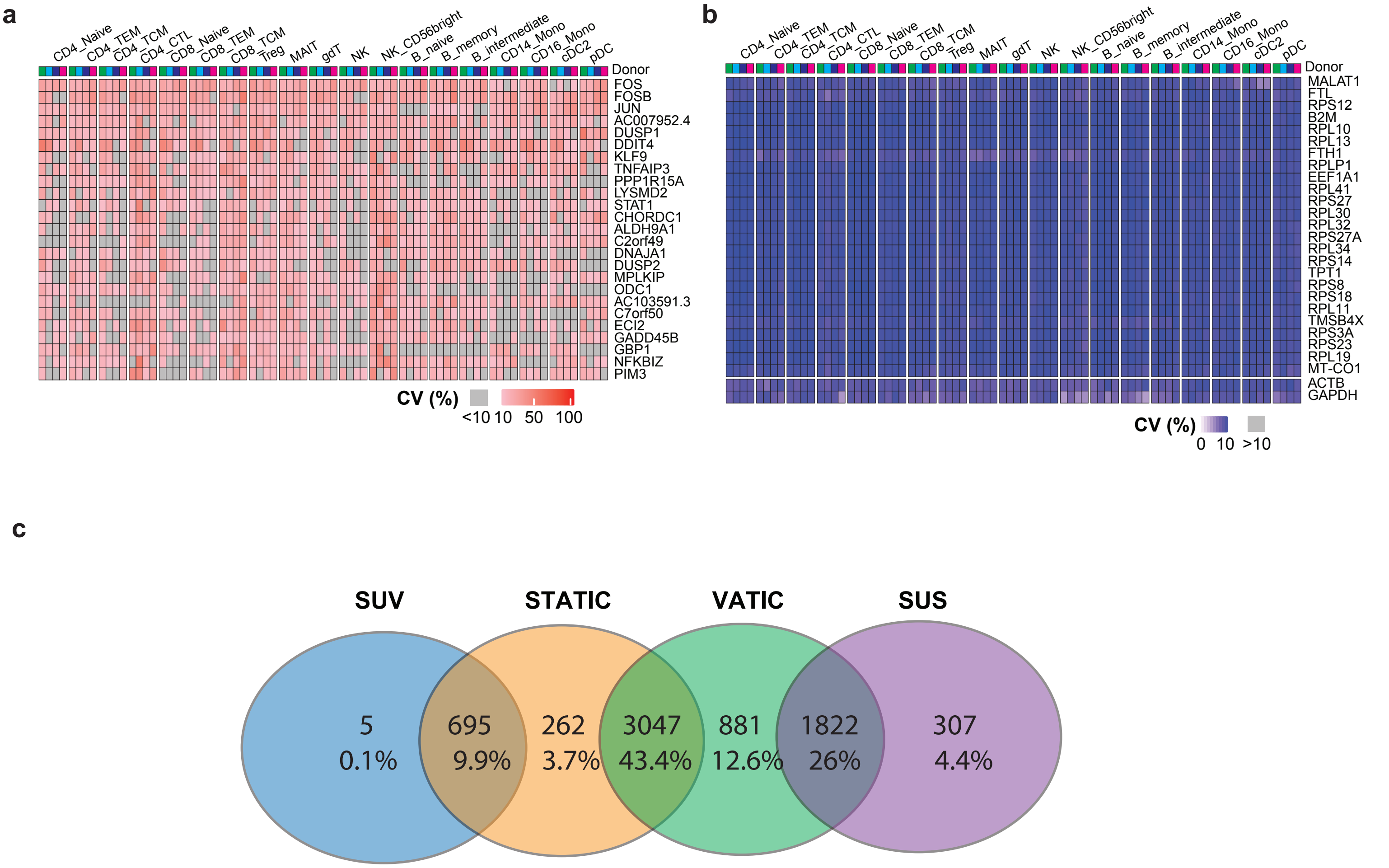

Supplementary Fig. 9: Longitudinally variable and stable genes across nineteen cell types. a Heatmap of coefficient of variation (CV) of the top 25 super-variable (SUV) genes. b Heatmap of CV of the top 25 super-stable (SUS) genes. **a,b** The CV for each of n=4 participants was measured on the corresponding 6 independent peripheral blood mononuclear cell (PBMC) samples. CVs of the housekeeping genes ACTB and GAPDH are also shown for comparison. **c** Venn diagram showing overlaps between SUV genes, stable across time in cell-types (STATIC) genes, variable across time in cell-types (VATIC) genes, and SUS genes. Adobe Illustrator (version 27.1.1; https://www.adobe.com/products/illustrator.html) was used to arrange panels and edit text.

Supplementary Figure. 10

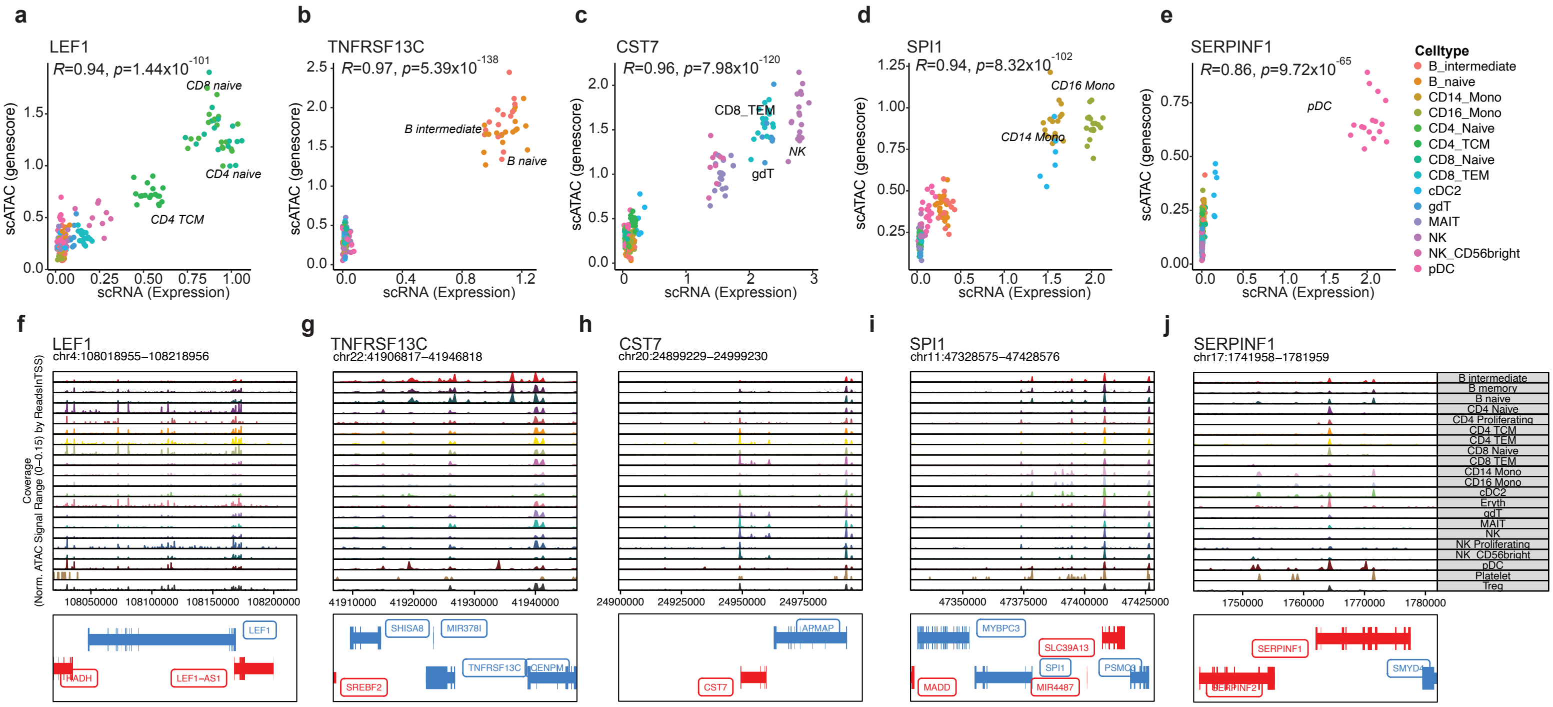

Supplementary Fig. 10: The five most correlated genes between expression in scRNA-seq data and gene score in scATAC-seq data. a-e Scatter plots between expression (pseudo-bulk intensity) in scRNA-seq data and gene score in scATAC-seq data of the five most correlated genes (LEF1, TNFRSF13C, CST7, SPI1 and SERPINF1). The Pearson correlation was calculated across 14 cell types in 18 independent peripheral blood mononuclear cell (PBMC) samples (n=252 data points). The p value (two sided) was calculated using R function “cor.test()”. **f-j** Open chromatin regions around the five most correlated genes in different cell types using ArchR21 visualization of scATAC-seq data. Adobe Illustrator (version 27.1.1; https://www.adobe.com/products/illustrator.html) was used to arrange panels and edit text.

Supplementary Figure. 11

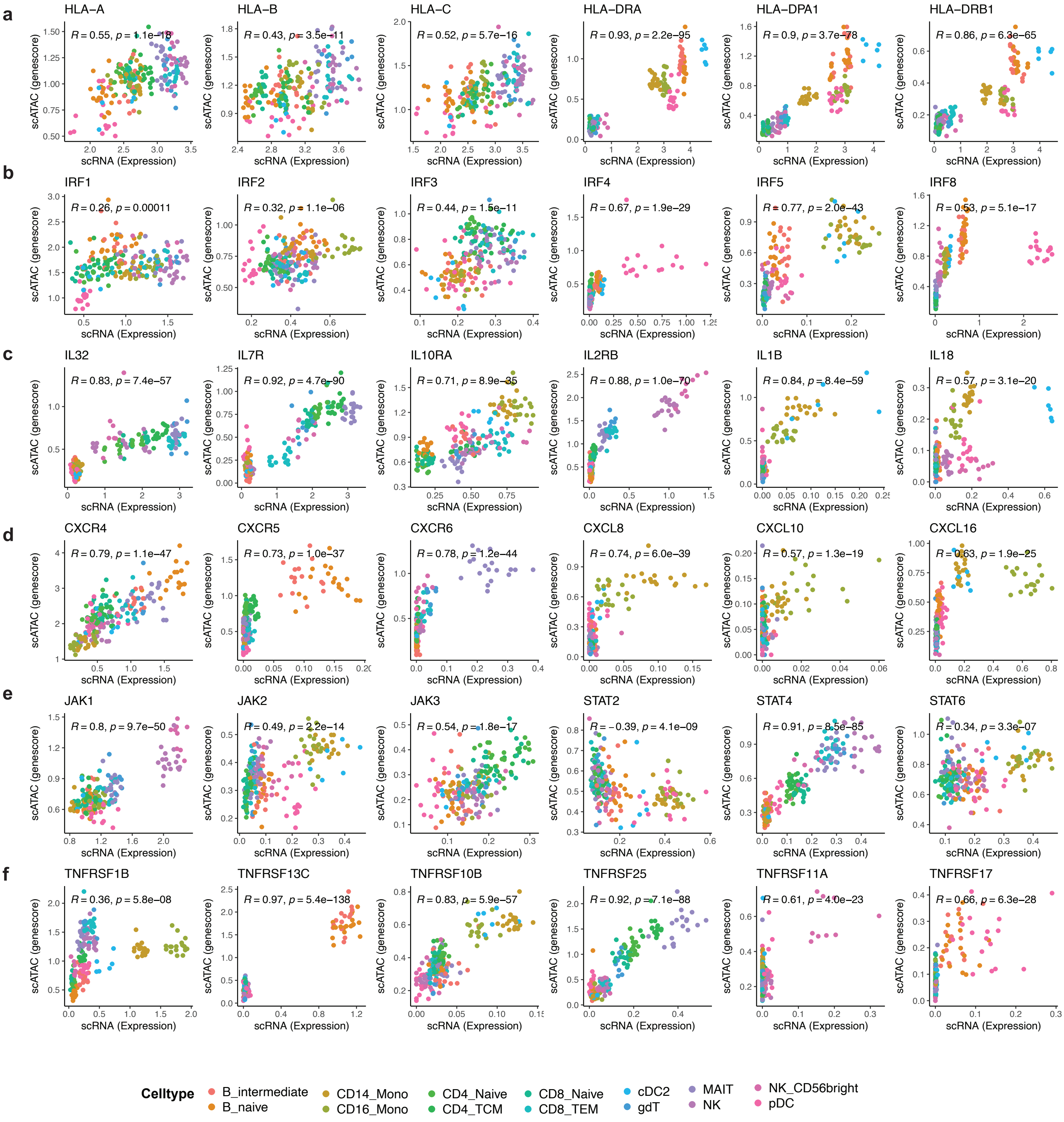

Supplementary Fig. 11: Correlations of six protein families between expresion in scRNA-seq data and gene score in scATAC-seq data. a Human leukocyte antigens (HLAs). **b** Interferon regulatory factors (IRFs). **c** Interleukins (ILs). **d** chemokine (C-X-C motif) receptor/ligand (CXCR/L) family. **e** Janus kinases (JAKs) and signal transducer and activator of transcription proteins (STATs). **f** Tumor necrosis factor receptor superfamily (TNFRSF). The Pearson correlation between expression (pseudo-bulk intensity) and gene score was calculated across 14 cell types in 18 independent peripheral blood mononuclear cell (PBMC) samples (n=252 data points). The p value (two sided) was calculated using R function “cor.test()”. Adobe Illustrator (version 27.1.1; https://www.adobe.com/products/illustrator.html) was used to arrange panels and edit text.

Supplementary Figure. 12

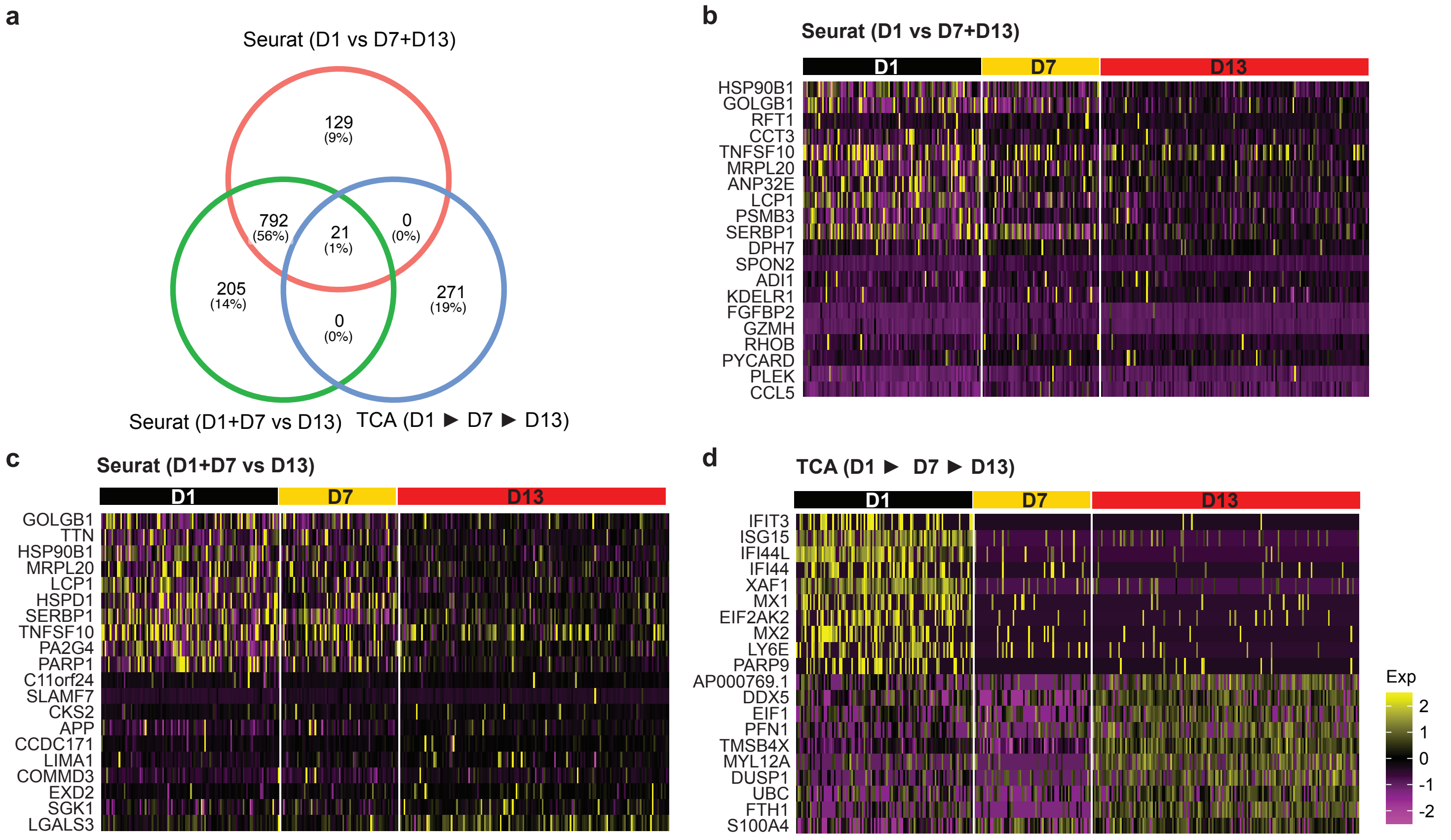

Supplementary Fig. 12: Comparison between time course analysis (TCA) and Seurat on longitudinal scRNA-seq data of a COVID-19 patient (COV-5). a Venn diagram for differential expression genes (DEGs) from TCA and DEGs from two runs of Seurat analyses: D1 versus D7+D13 or D1+D7 versus D13. **b-d** Top 10 up- and top 10 down-regulated genes from **b** Seurat D1 versus D7+D13 analysis, **c** Seurat D1+D7 versus D13 analysis and **d** TCA. The data was downloaded from CNP0001102[3]. Two-sided p values were calculated for individual genes based on either likelihood-ratio test (TCA) or Wilcoxon test (Seurat) and adjusted for multi-testing using Benjamini and Hochberg procedure. Genes with an adjusted p value <0.05 were identified as DEGs. Adobe Illustrator (version 27.1.1; https://www.adobe.com/products/illustrator.html) was used to arrange panels and edit text.
