# Supplementary material for: A comprehensive platform for analyzing longitudinal multi-omics data
Source: Nat Commun. 2023 Mar 27;14:1684. doi: 10.1038/s41467-023-37432-w (PMC10041512; doi:10.1038/s41467-023-37432-w)
Supplement: Supplementary file 3 — Editorial Assessment Report [file 41467_2023_37432_MOESM3_ESM.pdf]

nature portfolio

GUIDED OPEN ACCESS

nature methods | nature communications | communications biology

Contents of this report

1. **Manuscript details**: overview of your manuscript and the editorial team.
2. **Review synthesis**: summary of the reviewer reports provided by the editors.
3. **Editorial recommendation**: personalized evaluation and recommendation from all 3 journals.
4. **Annotated reviewer comments**: the referee reports with comments from the editors.
5. **Open research evaluation**: advice for adhering to best reproducibility practices.

About the editorial process

Because you selected the **Nature Portfolio Guided Open Access** option, your manuscript was assessed for suitability in three of our titles publishing high-quality work across the spectrum of methods research: ***Nature Methods***, ***Nature Communications***, and ***Communications Biology***. More information about Guided Open Access can be found here.

Collaborative editorial assessment

Your editorial team discussed the manuscript to determine its suitability for the Nature Portfolio Guided OA pilot. Our assessment of your manuscript takes into account several factors, including whether the work meets the **technical standard** of the Nature Portfolio and whether the findings are of **immediate significance** to the readership of at least one of the participating journals in the Nature Portfolio Guided Open Access methods cluster.

Peer review

Experts were asked to evaluate the following aspects of your manuscript:

- **Novelty** in comparison to prior publications;
- **Likely audience** of researchers in terms of broad fields of study and size;
- **Potential impact** of the study on the immediate or wider research field;
- **Evidence** for the claims and whether additional experiments or analyses could feasibly strengthen the evidence;
- **Methodological detail** and whether the manuscript is reproducible as written;
- Appropriateness of the **literature review**.

Editorial evaluation of reviews

Your editorial team discussed the potential suitability of your manuscript for each of the participating journals. They then discussed the revisions necessary in order for the work to be published, keeping each journal's specific editorial criteria in mind.

Journals in the Nature portfolio will support authors wishing to transfer their reviews and (where reviewers agree) the reviewers' identities to journals outside of Springer Nature.
If you have any questions about review portability, please contact our editorial office at guidedoa@nature.com.

Manuscript details

Tracking number	Submission date	Decision date	Peer review type
GUIDEDOA-21-00373	Dec 21, 2021	Feb 16, 2022	Single-blind

Manuscript title	Author details
PALM: a comprehensive platform for analyzing longitudinal multi-omics data	Xiao-jun Li<br>**Affiliation:** Allen Institute for Immunology

Editorial assessment team

**Primary editor**	**George Inglis**<br>Home journal: ***Communications Biology***<br>ORCID: **0000-0002-9069-5242**<br>Email: **george.inglis@us.nature.com**
**Other editors consulted**	**Lin Tang**<br>Home journal: ***Nature Methods***<br>ORCID: **0000-0002-6050-0424**<br><br>**Ilse Ariadna Valtierra Gutierrez**<br>Home journal: ***Nature Communications***<br>ORCID: **0000-0003-4128-5914**
**About your primary editor**	George received his PhD in Genetics and Molecular Biology from Emory University, where he studied mouse models of voltage-gated sodium channel dysfunction and epilepsy. He also has research experience in epigenomics and *in vitro* models of neuronal development. George joined the editorial team of *Communications Biology* in September 2020 and is based in the New York office.

Editorial assessment and review synthesis

**Editor's summary and assessment**	Here, the authors present PALM, a bioinformatic tool that can integrate bulk and single-cell data (including RNA-seq, proteomics, or ATAC-seq), along with clinical metrics like blood count, essentially streamlining the analysis of these varied data types. PALM is designed to integrate longitudinal data sets, to help users evaluate the stability of certain features in a dataset. As a demonstration of this workflow, the authors evaluate PBMC datasets from healthy participants or COVID-19 patients. They identify "STATIC" genes that are stable in terms of expression/chromatin profiles for each cell type, and resolve patient- and cell type-specific changes that might be linked to disease severity. Altogether, they conclude that PALM is a comprehensive platform to analyze longitudinal multi-omics data.<br><br>While the editors jointly decided to send this manuscript out to review based on the potential of the longitudinal analyses central to PALM, there were some concerns about the lack of benchmarking or limited complexity of the underlying samples, which prohibited further consideration by *Nature Methods*.
**Editorial synthesis of reviewer reports**	While Reviewers #4-5 find PALM to be of potential interest to the field, they raise several concerns regarding the degree of biological insight, underpinnings of the method, and its applicability to incomplete datasets or more complex samples (e.g. from another disease context, or tissue vs. PBMC samples). Reviewers #1-3 (co-reviewers) also comment on the limited technical advance of the method over tools like Seurat, potential issues in the central longitudinal analyses, and barriers for potential users of PALM. Taken together, these points supported the initial concerns from *Nature Methods*.<br><br>While *Nature Methods* is unable to offer a revision, *Nature Communications* would be interested in considering a revised manuscript that thoroughly demonstrates that PALM is capable of analysing single-cell datasets rigorously and specifically (Reviewers #1-3), allows users to define parameters (Reviewers #1-3), includes benchmarking to at least one alternative method (all reviewers), provides a benchmark with incomplete or smaller datasets (Reviewers #1-4), evaluates PALM on other complex datasets (Reviewer #5), and addresses technical issues with the analytical pipeline (all reviewers). Please do take into consideration that *Nature Communications* would not consider your manuscript further if your conclusions are weakened after addressing these concerns.<br><br>Alternatively, *Communications Biology* would be interested in considering a revised manuscript that at least qualifies any concerns about user-friendliness (even if the input format is not changed), includes benchmarking to at least one alternative method, discusses the potential of PALM to analyze incomplete or smaller datasets, and clarifies the overarching analytical pipeline.

Editorial recommendation

Nature Methods<br><br>Revision not invited	Neither the conceptual advance nor advance in performance demonstrated is sufficient for publication in *Nature Methods*.
Nature Communications<br><br>Major revisions with extension of the work	*Nature Communications* would be interested in considering a revised manuscript that thoroughly demonstrates that PALM is capable of analysing single-cell datasets rigorously and specifically (Reviewers #1-3), allows users to define parameters (Reviewers #1-3), includes benchmarking to at least one alternative method (all reviewers), provides a benchmark with incomplete or smaller datasets (Reviewers #1-4), evaluates PALM on other complex datasets (Reviewer #5), and addresses technical issues with the analytical pipeline (all reviewers). Please do take into consideration that *Nature Communications* would not consider your manuscript further if your conclusions are weakened after addressing these concerns.
Communications Biology<br><br>Major revisions	*Communications Biology* would be interested in considering a revised manuscript that qualifies concerns about user-friendliness (per Reviewers #1-3), includes benchmarking to at least one alternative method (all reviewers), discusses the potential of PALM to analyze incomplete or smaller datasets (Reviewers #1-4), and clarifies the analytical pipeline (all reviewers).

Next steps

**Editorial recommendation 1:**	Our top recommendation is to revise and resubmit your manuscript to *Communications Biology.* This option might be best if not all of the requested experimental revisions are possible/feasible at this time.
**Editorial recommendation 2:**	You may also choose to revise and resubmit your manuscript to *Nature Communications.* While we feel the additional required experiments are reasonable to achieve within a timeframe of 6 months, please keep in mind that *Nature Communications* would not consider your manuscript further if the conclusions are weakened after addressing these concerns.
**Note:**	As stated on the previous page *Nature Methods* is not inviting a revision at this time. Please keep in mind that the journal will not be able to consider any appeals of their decision through Guided Open Access.

<u>Revision</u>

To follow our recommendation, please upload the revised manuscript files using **the link provided in the decision letter**. Should you need assistance with our manuscript tracking system, please contact Adam Lipkin, our Nature Portfolio Guided OA support specialist, at guidedOA@nature.com.

Revision checklist

- ☐ Cover letter, stating to which journal you are submitting
- ☐ Revised manuscript
- ☐ Point-by-point response to reviews
- ☐ Updated Reporting Summary and Editorial Policy Checklist
- ☐ Supplementary materials (if applicable)

<u>Submission elsewhere</u>

If you choose not to follow our recommendations, you can still take the reviewer reports with you.

Option 1: Transfer to another Nature Portfolio journal
Springer Nature provides authors with the ability to transfer a manuscript within the Nature Portfolio, without the author having to upload the manuscript data again. To use this service, **please follow the transfer link provided in the decision letter.** If no link was provided, please contact guidedOA@nature.com.

> *Note that any decision to opt in to In Review at the original journal is not sent to the receiving journal on transfer. You can opt in to In Review at receiving journals that support this service by choosing to modify your manuscript on transfer.*

Option 2: Portable Peer Review option for submission to a journal outside of Nature Portfolio
If you choose to submit your revised manuscript to a journal at another publisher, we can share the reviews with another journal outside of the Nature Portfolio if requested. You will need to request that the receiving journal office contacts us at guidedOA@nature.com. We have included editorial guidance below in the reviewer reports and open research evaluation to aid in revising the manuscript for publication elsewhere.

Annotated reviewer reports

The editors have included some additional comments on specific points raised by the reviewers below, to clarify requirements for publication in the recommended journal(s). However, please note that all points should be addressed in a revision, even if an editor has not specifically commented on them.

Reviewers #1-3 information

**Expertise**	These reviewers have expertise in single-cell genomics.
**Editor's comments**	Please note that Reviewers #1-3 are co-reviewers, so their comments are identical. These reviewers highlighted barriers to user uptake of PALM, potential issues with the quality of the analysis, and limited technical advance over methods like Seurat, prohibiting further consideration by *Nature Methods*.

Reviewers #1-3 comments

**Section**	**Annotated Reviewer Comments**
**Remarks to the Author: Overall significance**	In the study "PALM: a comprehensive platform for analyzing longitudinal multi-omics data", Vasaikar et al. present a platform for analysing time series data generated from bulk and single-cell omics. The authors claimed that PALM is a comprehensive and simple-to-use platform, covering different topics in longitudinal analyses such as outlier detection, variable feature identification, inter/intra-donor variation assessment, multi-omics integration, etc. However, from my perspective, this work is far from being called a platform; instead, it is more like a patchwork of scripts by assembling different published functions/utilities into one repository without deep curations. It is not comprehensive, not simple-to-use, and importantly, not well-designed as is claimed in the manuscript.<br><br>1. PALM features the analyses of both bulk and single-cell omics data. However, several analyses of single-cell data (such as detecting features contributing towards donor variation) are achieved through pseudo-bulking the cell types. There are no dedicated functions/analyses to deal with single-cell-related problems. The other example is the quantification of intra-donor variation and the detection of outliers. While a CV-based approach can probably reflect the intra-donor variations over time in bulk data, in PALM this approach is also applied to single-cell datasets (such as the use of Z scores to determine outliers), which is dubious in terms of statistical rigour. A similar issue is in determining stable versus variable features – several thresholds are simply deployed to obtain this distinction without rigorous statistical motivation. This might be useful in an individual project to explore the data of interest, but is not sufficient for a service provided by a platform.<br>**Justification of PALM's analytical pipeline would be necessary for further consideration at *Communications Biology*. However, for further**

consideration at *Nature Communications*, it would also be essential to thoroughly demonstrate that PALM's model is capable of analysing single-cell datasets rigorously and specifically; for instance, with ground truth-based benchmarking.

2. PALM is not an easy-to-use platform.
2.1. Many parameters are hard coded in PALM. The users have to adjust their data greatly to accommodate the platform, like changing their metadata column names, matching the information, etc. In addition, most functions are designed in a customized manner for the authors of the manuscript, rather than suiting potential users. One of the examples can be found in the PALM repo, https://github.com/aifimmunology/PALM/blob/main/R/avgExpCalc.R, where the group.by parameter (line 19) is strictly hard coded in the code while soft coded in the interface (line 14). The authors seem not ready for pushing PALM out as a light-weight platform.

For further consideration at *Nature Communications*, we would expect you to address this concern by allowing the potential users to define relevant parameters. *Communications Biology* would also encourage this change, though this point could also be addressed by qualifying any claims of user-friendliness throughout the text.

2.2. Walking through the PALM tutorial (https://github.com/aifimmunology/PALM#introduction), the functions in PALM are not well integrated into an entire ecosystem. For example, after finding the donor-contributing features, there is no such a function to explore these features; instead, it is a step-by-step guide in how to write analytical codes to display and visualise them. It indeed feels like walking through a series of scripts in the code repo of a project, instead of a platform.

2.3. There are several other points that make PALM hard-to-use, including: i) The package is not directly available in CRAN or Bioconductor. ii) Metadata (e.g., the names, text format, delimiter and many others) have to be arranged in a way that is exactly as the authors' data. iii) Tutorials are filled with codes with almost no explanations in each step to guide the users. iv) There are limited flexible options to perform works at the user end. v) All utilities are function-based rather than class-based (S3/4 for example), making PALM a rough collection of functions.

Both *Nature Communications* and *Communications Biology* would strongly recommend that the package be made available in CRAN or Bioconductor.

3. PALM is not comprehensive in terms of tackling different kinds of tasks in longitudinal data. For example, during the intra-donor analysis, each participant is associated with several time points. What if the experimental design is not balanced, say, some participants have fewer time points? Will such a CV-based analysis still be useful? What if some time points have several technical replicates? Should these replicates be collapsed before the analysis? Or the CV analysis can be applied directly? All these are directly related to Comment 2, that is, PALM cannot

	accommodate different datasets from the users, being only suitable to the authors' data in a relatively strict sense. The lack of comprehensive longitudinal analysis features makes it underwhelming, as the key selling point of PALM is that this is first-of-its-kind platform to analyse longitudinal multiomics data. Importantly, the presented longitudinal analysis features are again just using previously published tools.<br>**This point was also raised by Reviewer #4. Performing an actual analysis to demonstrate this point using smaller datasets (e.g. with some samples missing time points) would be essential for further consideration at *Nature Communications* and *Communications Biology*.**<br><br>4. Many analyses in PALM are not novel, such as the variance decomposition. DE analyses, and circos plot. Even without novel analysis provided in PALM, the authors should at least provide a quick interface to use the external tools. For example, since the authors use the linear mixed model to assess the random effects during variance decomposition, how about adding dedicated methods to extract the deviated donors, instead of just showing a code guide to plot these genes and locate the donors (https://github.com/aifimmunology/PALM#plot-the-top-variables)? I also expect that more analytical workflows will be added in PALM to handle more practical problems in longitudinal analysis.<br><br>5. The authors claim that this is a first-of-its-kind platform to analyse single cell longitudinal multiomics data. There are however various tools out there that are capable of doing this. The most notable example here is Seurat which the authors actually extensively use for both data object architecture and analysis features. Seurat offers extensive integrative approaches for multiple modalities. Amongst other options, it also offers longitudinal data analysis through for example the MAST, which is again the same as what the authors use.<br>**This point was also hinted at by Reviewers #4-5. Please benchmark PALM to at least one other existing method, for further consideration at *Nature Communications* and *Communications Biology*.**

Reviewer #4 information	
**Expertise**	This reviewer has expertise in multi-omic methods and machine learning.
**Editor's comments**	This reviewer finds PALM to be a valuable tool for evaluating longitudinal datasets, but highlights the need for clearer descriptions of the underlying method (see Major Concern #1), whether it is applicable to smaller datasets, and its performance relative to other methods.

Reviewer #4 comments

Section	Annotated Reviewer Comments
**Remarks to the Author: Overall significance**	**Overview summary**<br>The authors developed a platform named PALM to conduct an integrative and comprehensive analysis using longitudinal bulk and single-cell multi-omics datasets. Applying PALM, they can perform variance decomposition, identify STATIC features, determine the abnormal dataset outliers. Overall, PALM provides a unique and comprehensive insight to understand the biological questions.<br><br>**Major concerns**<br>1. The authors demonstrate various functions within the PALM platform. However, I got quite confused what is the input for PALM. Does PALM take Seurat object as an input or does it take the raw matrices as inputs? Also, the analytical pipeline of PALM is a little bit ambiguous. Can you explain the pipeline of PALM?<br>**For the sake of reproducibility, please be sure to clarify the underlying PALM pipeline. Please note that the Methods section in Nature Portfolio journals does not have a strict word limit.**<br><br>2. In this manuscript, the authors provide a lot of data including 4 scRNA-seq samples with 10 different time points each. I am wondering whether we can apply the PALM platform to small datasets, like only 1 scRNA-seq sample with 2 or 3 different time points? Specifically, can the detection of abnormal timepoints function work successfully?<br>**This point was also raised by Reviewers #1-3. Performing an actual analysis to demonstrate this point would be essential for further consideration at *Nature Communications* and *Communications Biology*.**<br><br>3. An important finding in this manuscript is STATIC identification. I wonder what is the major difference between STATIC and conserved differentially

expressed genes/features across time points? Specifically, if you run an alignment algorithm like Seurat or LIGER using all different time points and different samples, and you identified the conserved differentially expressed genes for each cluster, I am wondering how many overlaps between the STATIC genes and the conserved differentially expressed genes?

The need for further benchmarking is also echoed by Reviewer #5. Please benchmark PALM to at least one other method for further consideration at *Nature Communications* and *Communications Biology*.

Minor concerns:

1. On page 3 lines 101-105, there are lots of longitudinally variable and stable genes. I wonder what are the biological insights to determine the longitudinally variable and stable genes? Are you considering this in the developmental processes or drug treatment/perturbation processes?

Please elaborate on the underlying biological insights in the Discussion, for further consideration at *Nature Communications* and *Communications Biology*.

2. For the scRNA-seq method part, it is a little bit unclear how many samples and how many time points were used for sequencing. Also, I am confused about why you run your scRNA-seq QC (nFeature, percent.mt, etc.) after you conduct the label transferring?

It would be essential to address this concern for further consideration at *Nature Communications,* preferably with additional analyses or test cases.

3. The description of your linear mixed model is not clear. You have considered donor, time, and cell type as random effects but not mentioned what variables you use for fixed effects. Could you provide more details?

Reviewer #5 information

**Expertise**	This reviewer has expertise in immunogenomics, bioinformatics, and single cell biology.
**Editor's comments**	This reviewer echoes several concerns from Reviewers #1-4, and also emphasizes the need for further demonstration of how PALM could be applied to complex data types (like tissue samples).

Reviewer #5 comments

**Section**	**Annotated Reviewer Comments**
**Remarks to the Author: Overall significance**	In their submitted work entitled "PALM: a comprehensive platform for analyzing longitudinal multi-omics data", the authors developed PALM, a platform to analyze longitudinal bulk and single-cell multi-omics data. They applied PALM to their own and public datasets to show the utility of this approach. PALM is a simple and useful tool. However, the paper suffers from several major limitations.<br><br>1. The authors applied PALM to analyze scRNA-seq and scATAC-seq datasets and their analyses were mainly focused on PBMCs. The tissue samples may have different cell compositions from blood samples, but they were not included. Currently, huge amounts of scRNA-seq and scATAC-seq data have been generated on tissue samples from many published studies, the authors are suggested showing the performance of PALM on tissue samples.<br>**This point would be necessary for further consideration at *Nature Communications*, but should be mentioned as a limitation for *Communications Biology*.**<br><br>2. Likewise, the authors utilized PALM to analyze data generated on samples from healthy donors but did not show its performance on data generated from subjects with other conditions such as cancer, developmental diseases, or other genetic diseases, and adding such examples are encouraged in order to show the potentially broad application of PALM.<br>**Given that SARS-CoV-2 datasets were already incorporated into the manuscript, this point would not be necessary for further consideration at *Communications Biology*. However, for further consideration at *Nature Communications*, we would expect your revision to provide additional applications of PALM to datasets from other diseases.**<br><br>3. There are other existing tools that have been already developed to integrate

single-cell multi-omics data (e.g., scRNA-seq and scATAC-seq data integration) and the authors are suggested comparing the performance of PALM with other tools.

The need for further benchmarking is also echoed by other reviewers. Please benchmark PALM to at least one other method for further consideration at *Nature Communications* and *Communications Biology*.

4. Can PALM be used to analyze scTCR/BCR-seq data? scTCR/BCR-seq approach is commonly used in biomedical research. The same for spatial transcriptomics /proteomics data.

We would strongly recommend you to include such benchmarking for further consideration at *Nature Communications.* In contrast, for *Communications Biology*, while direct benchmarking would not be necessary for scTCR- or scBCR-seq data, we would ask that you comment on this point in the Discussion.

5. To better understand the SUV, SUS, STATIC gene lists, analyses across different tissue types, cell types (e.g., normal, inflammatory, or malignant cell types and states), and across different disease conditions are necessary.

This point would be necessary for further consideration at *Nature Communications*, but (as noted in Point #2) not *Communications Biology*.

6. The key data quality control steps warrant further check as some of the key steps of data processing (such as batch effects evaluation, correction, doublet removal, etc.) are not provided in the Methods.

7. It is unclear how PALM defines cell types/states, and cell types/states identified in PBMCs are fewer (not comprehensive) than previously reported.

8. Importantly, data analysis modules included in PALM are kind of basic. To comprehensively analyze the multi-omics data, deep profiling approach would be needed.

Open research evaluation

Guidelines for Transparency and Openness Promotion (TOP) in Journal Policies and Practices ("TOP Guidelines")

The recommendations and requests in the table below are aimed at bringing your manuscript in line with common community standards as exemplified by the TOP Guidelines. While every publisher and journal will implement these guidelines differently, the recommendations below are all consistent with the policies at Nature Portfolio. In most cases, these will align with TOP Guidelines Level 2.

FAIR Principles

The goal of the recommendations in the table below related to **data or code** availability is to promote the FAIR Guiding Principles for scientific data management and stewardship (*Scientific Data* **3**: 160018, 2016). The FAIR Principles are a set of guidelines for improving 4 important aspects of digital research objects: **F**indability, **A**ccessibility, **I**nteroperability and **R**eusability.

ORCID

ORCID is a non-profit organization that provides researchers with a unique digital identifier. These identifiers can be used by editors, funding agencies, publishers, and institutions to reliably identify individuals in the same way that ISBNs and DOIs identify books and articles. Thus the risk of confusing your identity with another researcher with the same name is eliminated. **The ORCID website** provides researchers with a page where your comprehensive research activity can be stored.

Springer Nature collaborates with the ORCID organization to ensure that your research contributions (as authors and peer reviewers) are correctly attributed to you. Learn more at https://www.springernature.com/gp/researchers/orcid

Data availability
**Data Availability Statement**
Thank you for including a Data Availability statement. While you have included some important information, the editors have noted that some details appear to be missing.The Data Availability Statement should be as detailed as possible and include accession codes or other unique IDs for deposited data, information about where source data can be found, and specify any restrictions to data access that may apply. At a minimum, the statement should indicate that data are available upon request and explain how data access can be granted. If data access is not possible, the reasons for this must be made clear in the Data Availability Statement.<br><br>More information about the Nature Portfolio data availability policy can be found here.<br><br>**Please clarify in the Data Availability Statement where source data for Fig 1b, 1d, and 1g-i can be obtained.**<br><br>More information about formatting Data Availability Statements can be found here.
**Mandatory data deposition**
Most scientific journals, including all Nature Portfolio journals, require that any newly-generated sequence data must be made publicly available before publication. There are some exceptions allowed for sensitive clinical data, but this should be discussed with the editor. All data must be deposited in a community-approved repository and accession codes/unique IDs must be included within the Data Availability Statement in the manuscript.<br><br>Examples of appropriate public repositories are listed below:<br>• GenBank (all DNA sequence data)<br>• Sequence Read Archive (high-throughput sequence data)<br>• Gene Expression Omnibus (Microarray or RNA sequencing data)<br><br>**Please provide a reviewer token for GSE190992, or make this dataset publicly available.**
More information on mandatory data deposition policies at the Nature Portfolio can be found at http://www.nature.com/authors/policies/availability.html#data<br><br>Please visit https://www.springernature.com/gp/authors/research-data-policy/repositories/12327124 for a list of approved repositories for various data types.
**Other data requests**
In line with community standards regarding open research, Springer Nature strongly supports data sharing and believes that all datasets on which the conclusions of the paper rely should be available to readers. We encourage authors to ensure that their datasets are either deposited in publicly available repositories (where available and appropriate) or presented in the main manuscript or additional supporting files whenever possible.<br><br>To learn more about data sharing and recommended data repositories, please see https://www.springernature.com/gp/authors/research-data-policy/repositories/12327124

All source data underlying the graphs and charts presented in the main figures must be made available as Supplementary Data (in Excel or text format) or via a generalist repository (eg, Figshare or Dryad). This is mandatory for publication in a Nature Portfolio journal, but is also best practice for publication in any venue.

The following figures require associated source data: Fig 1b, 1d, and 1g-i

Data citation

Please cite (within the main reference list) any datasets stored in external repositories that are mentioned within their manuscript. For previously published datasets, we ask that you cite both the related research article(s) and the datasets themselves. For more information on how to cite datasets in submitted manuscripts, please see our data availability statements and data citations policy: https://www.nature.com/documents/nr-data-availability-statements-data-citations.pdf

Citing and referencing data in publications supports reproducible research, by increasing the transparency and provenance tracking of data generated or analysed during research. Citing data formally in reference lists also helps facilitate the tracking of data reuse and may help assign credit for individuals' contributions to research. A number of Springer Nature imprints are signatories of the Joint Declaration on Data Citation Principles, which stress the importance of data resources in scientific communication.

Thank you for depositing your dataset in a public repository. In addition to providing the link within the Data Availability statement, we ask that you also cite the dataset in the main reference list.

Code availability and citation

Thank you for making your custom code available via Github. Upon publication, Nature Portfolio journals consider it best practice to release custom computer code in a way that allows readers to repeat the published results. Code should be deposited in a DOI-minting repository such as Zenodo, Gigantum or Code Ocean and cited in the reference list following the guidelines described in our policy pages (see link below). Authors are encouraged to manage subsequent code versions and to use a license approved by the open source initiative.

See here for more information about our code availability policies:

Ethics

We believe that research that involves the use of clinical, biomedical or biometric data from human participants must only be carried out with the explicit consent of those whose data are involved. Consent must be obtained without any form of coercion and with participants' explicit understanding of the purpose for which their data will be used.

Because your study includes human participants, confirmation that all relevant ethical regulations were followed is needed for publication in any Springer Nature journal, and that informed consent was obtained. This must be stated in the Methods section, including the name of the board and institution that approved the study protocol.

Further details about the Nature Portfolio policy can be found at https://www.nature.com/commsbio/editorial-policies/ethics-and-biosecurity

Reporting & reproducibility

We believe that research publications should adhere to high standards of transparency and robustness in their methods and results. This, in turn, supports the principle of reproducibility, which is a foundation of good research, especially in the natural sciences. All data that support the conclusions drawn must be presented in the manuscript unless they are published elsewhere.

Nature Portfolio journals do not allow statements of "data not shown". Please remove these statements or provide the relevant data.

Line 473: Unpublished dataset from Talla et al 2021 (GSE173590)

We believe that research publications should adhere to high standards of transparency and robustness in their methods and results. This, in turn, supports the principle of reproducibility, which is a foundation of good research, especially in the natural sciences.

The Methods section should contain sufficient detail such that the work could be repeated. It is preferable that all key methods be included in the main manuscript, rather than in the Supplementary Information. Please avoid use of "as described previously" or similar, and instead detail the specific methods used, with appropriate attribution.

- **Lines 203-214:** PMBC isolation cryopreservation methods

Please note that Nature Portfolio journals allow unlimited space for Methods.

Materials availability

Oligo sequences, concentrations of antibodies, and sources of cell lines must be included in the Methods (these can also be provided in a main Table and cited in the Methods). Please see the Nature Portfolio policy page for further details:
https://www.nature.com/commsbio/editorial-policies/reporting-standards#availability-of-materials

Statistical reporting

Wherever statistics have been derived (e.g. error bars, box plots, statistical significance) figure legends should provide and define the n number (i.e. the sample size used to derive statistics) as a precise value (not a range), using the wording "n=X biologically independent samples/animals/cells/independent experiments/n= X cells examined over Y independent experiments" etc. as applicable. The figure legends must also indicate the statistical test used. Where appropriate, please indicate in the figure legends whether the statistical tests were one-sided or two-sided and whether adjustments were made for multiple comparisons. For null hypothesis testing, please indicate the test statistic (e.g. F, t, r) with confidence intervals, effect sizes, degrees of freedom and P values noted.

All error bars need to be defined in the figure legends (e.g. SD, SEM) together with a measure of centre (e.g. mean, median). For example, the legends should state something along the lines of "Data are presented as mean values +/- SEM" as appropriate. All box plots need to be defined in the legends in terms of minima, maxima, centre, bounds of box and whiskers and percentile.

For examples of expected description of statistics in figure legends, please see the following:
https://www.nature.com/articles/s41467-019-11636-5 or
https://www.nature.com/articles/s41467-019-11510-4.

When describing results as "significant" in the main text, please include details about the statistical test used and provide an exact p-value, rather than a signifcance threshold.
Please note that statistics such as error bars significance and p values cannot be derived from n<3 and must be removed in all such cases.
We strongly discourage deriving statistics from technical replicates, unless there is a clear scientific justification for why providing this information is important. Conflating technical and biological variability, e.g., by pooling technically replicated samples across independent experiments is strongly discouraged.
To improve reproducibility of your analyses, please provide details regarding your treatment of outliers.
To improve reproducibility of your analyses, please detail the methods used for data fitting and provide a rationale for this approach.
