# Supplementary material for: A comprehensive platform for analyzing longitudinal multi-omics data
Source: Nat Commun. 2023 Mar 27;14:1684. doi: 10.1038/s41467-023-37432-w (PMC10041512; doi:10.1038/s41467-023-37432-w)
Supplement: Supplementary file 4 — Description of Additional Supplementary Files [file 41467_2023_37432_MOESM4_ESM.docx]

**Inventory of Supporting Information**

**Supplementary Figure 1-12**

**Supplementary Data 1-8**

**Source Data**

**Supplementary Data**

**Supplementary Data 1:** Longitudinal bulk data of n=6 healthy participants over 10 weeks, including complete blood count (CBC, 60 independent samples), flow cytometry-based cell frequencies (24 independent samples), and plasma proteomics data (1156 proteins, 60 independent samples).

**Supplementary Data 2:** PALMO results on longitudinal plasma proteomics data of n=6 healthy participants (60 independent samples), including results from variance decomposition analysis (VDA, 2a), coefficient of variation profiling (CVP, 2b-d), and outlier detection analysis (ODA, 2e-g).

**Supplementary Data 3:** PALMO results on longitudinal scRNA-seq data (24 independent samples) data and scATAC-seq data (18 independent samples) of n=4 healthy participants, including cell types identified, results from variance decomposition analysis (VDA), stability pattern evaluation across cell types (SPECT), and pathway enrichment analysis.

**Supplementary Data 4:** Variance decomposition analysis (VDA) results on T-cell receptor (TCR) repertoires of n=4 systemic sclerosis donors (12 independent samples). The data was downloaded from GSE156980 (Servaas et al., J. Autoimmun. **117**, 102574 (2021)).

**Supplementary Data 5:** Top 220 stable across time in cell-types (STATIC) genes, pathways enriched with STATIC genes, and Pearson's correlation between gene expression in scRNA-seq data and gene score in scATAC-seq data.

**Supplementary Data 6:** Top 304 stable across time in cell-types (STATIC) genes observed in 25 cell types from mouse brain dataset GSE129788 (Ximerakis et al., Nat. Neurosci. **22**, 1696-1708 (2019)) and their overlap with the corresponding marker genes of the same cell types.

**Supplementary Data 7:** List of up- or down-regulated genes observed from time course analysis (TCA) on longitudinal scRNA-seq data of n=4 COVID-19 patients (CNP0001102; Zhu et al., Immunity **53**, 685-696 (2020)) and the corresponding enriched pathways.

**Supplementary Data 8:** List of reagents and resources used in our longitudinal study of n=6 healthy participants.
